# New Appliances

**Published:** 1889-06

**Authors:** 


### EDITORIAL.

### New Appliances.

Those who have done much in the way of mounting gold crowns and bridge-work understand and appreciate the difficulty oftentimes experienced in removing the bands and crowns during the process of fitting and adjusting. This part of the work has been rendered easy by the use of a little device of Dr. C. H. Rosenthall, of Cincinnati. It consists of a staple-form clutch, the points of which catch the edge of the crown or band at or under the gum margin; a screw shaft then passes through the crown part of the staple working through a screw-nut on the under side of the staple, and as the screw is operated its end comes in contact with the root or crown to which the substitute is being adjusted, the band or crown is removed from the root without the least jar or strain upon it. Only those who have used it can appreciate the great relief its use affords.

It will very soon be on sale at the dental depots or it can be obtained of Dr. R.

Another very convenient and useful appliance, especially useful in crown and bridge-work, is a Lilliputian rolling mill, also obtained through Dr. Rosenthall; of this he does not claim to be the inventor though it was made at his suggestion. The rollers are two inches long and an inch and a half in diameter, set in a strong frame; the whole weighing about twenty pounds. It is as efficient as a larger mill for working small quantities of metal, even to an ounce. An interesting point about it is that it is furnished ready for operation for $15.00.

